# Delignification efficiency of various types of biomass using microwave-assisted hydrotropic pretreatment

**DOI:** 10.1038/s41598-022-08717-9

**Published:** 2022-03-16

**Authors:** Dawid Mikulski, Grzegorz Kłosowski

**Affiliations:** Department of Biotechnology, Kazimierz Wielki University, ul. K. J. Poniatowskiego 12, 85-671 Bydgoszcz, Poland

**Keywords:** Biotechnology, Plant sciences

## Abstract

The use of a method of an effective delignification of lignocellulosic biomass is a key stage of designing processes of its microbiological conversion e.g. for the purposes of the production of cellulosic ethanol. The study was aimed at evaluating the effectiveness of microwave-assisted hydrotropic pretreatment using sodium cumene sulfonate (NaCS) for the delignification of pine and beech chips and wheat straw. Research results presenting the impact of process parameters of microwave-assisted hydrotropic delignification confirm a high effectiveness of this method of pretreatment of lignocellulosic biomass. The observed effects included changes in the composition of the biomass and an increased susceptibility of cellulose to the subsequent enzymatic hydrolysis. The use of microwave heating combined with an addition of hydrotrope of 40% w/v NaCS and 117 PSI for 60 min enabled a reduction of the absolute concentration of lignins by 36.58% in pine chips, by 57.68% in beech chips, and by 74.08% in wheat straw. After enzymatic hydrolysis was conducted, the highest concentration of glucose: 463.27 ± 11.25 mg glucose/g (hydrolysis yield 46.76 ± 1.14%) was obtained from the wheat straw, while 327.70 ± 22.15 mg glucose/g (hydrolysis yield 35.13 ± 2.37%) was acquired from the beech chips, and only 50.77 ± 0.75 mg glucose/g (hydrolysis yield 6.63 ± 0.10%) was obtained from the pine chips. Microwave-assisted hydrotropic delignification in the optimum process conditions additionally allows a complete removal of hemicellulose from biomass, which improves the effectiveness of enzymatic hydrolysis. Due to a significant reduction of lignin and hemicellulose concentration in biomass, cellulose—which is susceptible to enzymatic hydrolysis and a source of carbon in biosynthesis processes—becomes the main biomass component.

## Introduction

Structural polysaccharides (cellulose and hemicellulose), which are the main components of lignocellulosic biomass, can be an inexpensive and easily available source of carbon used in biosynthesis processes; however, they must be susceptible to enzymatic degradation. An increase in the susceptibility of cellulose and hemicellulose to enzymatic hydrolysis is achieved during pretreatment aimed at reducing crystalline areas in cellulose, increasing the porosity of cellulose and hemicellulose and, primarily, removing lignins^1,2^. An assessment of the effectiveness of pretreatment methods involves an analysis of the concentration of by-products of the dehydration of sugars, i.e. furfural from xylose, or 5-(hydroxymethyl)furfural (5-HMF) from glucose. These compounds show features of inhibitors and have a negative effect on the cellular metabolism of microorganisms and the catalytic activity of many enzymes^3,4^. Additionally, a partial modification of the lignin structure as a result of the use of various pretreatment methods may generate an increased number of hydrogen and hydrophobic interactions between lignin and cellulases, which can significantly limit the catalytic effectiveness of cellulases. Both lignins and products of their decomposition or modification may bind with cellulases, while their binding with enzymes may be an effect of hydrophobic, electrostatic interactions and the creation of hydrogen bonds^5,6^. The removal of lignins from plant biomass is a key condition enabling the acquisition of the structural polysaccharides fraction susceptible to enzymatic hydrolysis. This is facilitated by the specific structure in which cellulose fibres and hemicellulose are surrounded by lignin, which lowers the susceptibility of the mentioned polysaccharides to the action of hydrolytic enzymes and physical and chemical factors^7,8^. This phenomenon is also connected with the hydrophobic nature of lignins, which are a phenolic heteropolymer composed of monolignol units, i.e. syringyl (two methoxyl groups), guaiacyl (one methoxyl group) and p-hydroxyphenylpropane^9^. The molecular structure of lignins determines the high resistance of plant biomass to degradation processes, which is a factor limiting its use as a raw material in bioconversion processes e.g. for cellulosic bioethanol production. Lignins limit susceptibility of biomass to hydrolysis by producing a physical barrier between polysaccharides and hydrolytic enzymes (lignin-polysaccharide complexes) and by binding enzymes and their inhibition^10^. The growing demand for renewable sources of carbon used in microbiological syntheses is an impulse for searching for effective methods of the delignification of biomass of various origins. There are many lignin removal methods and they are marked by different effectiveness in relation to a given type of biomass and a differing profitability resulting from the specificity and parameters of the operations and unit processes involved^11^. The alkaline method using sodium hydroxide is the basic and best-described method enabling the breaking of the bonds between lignin and hemicellulose for the purposes of effective delignification. The process of delignification using NaOH is composed of several stages and begins with the breaking of phenolic bonds α-O-4, β-O-4, β-O-5, and β-β in lignin. The non-phenolic β-O-4 bonds and carbon–carbon bonds in lignin are then broken and, additionally, carbohydrates undergo degradation^12^. Alkaline delignification is often aided with other pretreatment methods, such as the use of ultrasounds or steam explosion^13–15^. Another strategy used during biomass delignification is the use of substances increasing the solubility of hydrophobic compounds (including lignins) in aqueous solutions. Such compounds include organic solvents such as ethanol, ionic liquids, deep eutectic solvents and hydrotropes^16–19^. Hydrotropes (sodium and potassium salts of benzoic and aryl sulfonic acids with a substituted alkyl group) are compounds with a structure and properties similar to those marking surfactants. However, in contrast to surfactants, hydrotropes do not produce micelles due to their smaller hydrophobic structure. The amphiphilic structure of hydrotropes predisposes them to be used as a factor able to effectively lower the surface tension of liquids and increase lignin solubility during pretreatment. The possibility to precipitate lignins by diluting hydrotrope with water is an additional advantage of using hydrotropes in delignification processes. The thus-produced lignins can then be used for the production of derivatives or polymers, while the hydrotrope solution can be used again after concentration for lignocellulose pretreatment. The possibility to recirculate hydrotropic solution improves the economy of the delignification process^20–23^. The effectiveness of the use of substances such as hydrotropes, deep eutectic liquids and ionic liquids in the process of delignification, depends on the type of biomass (composition of lignins) and process conditions. To intensify the lignin removal process, microwave radiation can be used^24^. Apart from an increase in medium temperature, the use of microwaves, i.e. electromagnetic radiation of 0.3–300 GHz, is marked by the oscillatory movement of water dipoles, thus stimulating the degradation of the lignocellulosic biomass^25^. Additional advantages of the use of microwave radiation in biomass pretreatment include the rate of biomass temperature increase and close control of the course of this operation, as well as the possibility to use various chemical substances such as mineral acids, deep eutectic liquids or hydrotropes during its course. Microwave-assisted pretreatment of biomass combined with the use of inorganic acids or hydroxides makes it possible to obtain raw material with a lowered content of lignins and hemicellulose. However, the degree of degradation of structural polysaccharides depends on the process parameters of the microwave-assisted pretreatment. The main factors deciding about the concentration of sugars in hydrolysates include the type of reagent and time^26,27^. The effectiveness of the use of microwave radiation in lignocellulosic biomass pretreatment was confirmed for food industry waste, cereal straw, distillery stillages, and exotic plant biomass^28–32^.

The necessity to use lignocellulosic biomass effectively as an inexpensive and renewable source of carbon for example in the process of the production of second generation ethanol requires the development of effective ways of the delignification of various types of biomass. From among plant raw materials, the use of waste material from the timber industry such as pine and beech chips, categorised respectively as softwood and hardwood biomass, is limited in biosynthesis processes. This results from the high resistance of this type of biomass to degradation processes. So far, no research has been carried out on the impact of the process parameters of microwave-assisted hydrotropic pretreatment on the course and effects of the delignification of softwood, hardwood and non-wood biomass. The study was aimed to determine the impact of changeable conditions of microwave-assisted hydrotropic delignification using NaCS on the effectiveness of the delignification of biomass of different origins and changes in the composition of lignocellulose as well as its susceptibility to hydrolysis involving cellulases. Tests were conducted with the microwave generator at a constant power of 600 W, but with a variable concentration of NaCS (10, 20, 40%, w/v), time (10, 30, 60 min) and pressure (39, 78, 117 PSI). In view of the high extractability of hydrophobic substances from lignocellulosic biomass, we chose sodium cumene sulfonate (NaCS) as a hydrotrope for our study^33^. NaCS, being an industrial reagent, is easily available in international trade, which was an additional reason behind our choice. The pre-treated biomass was evaluated for its extractability, lignocellulose composition (determination of the content of cellulose, hemicellulose, and lignins) and susceptibility to enzymatic degradation involving cellulosic enzymes. An additional novel aspect was the assessment, for the first time, of the biomass delignification degree, taking into account the level of extraction of its components during microwave-assisted hydrotropic pretreatment.

## Results

### Biomass pretreatment

**Figure 1:**
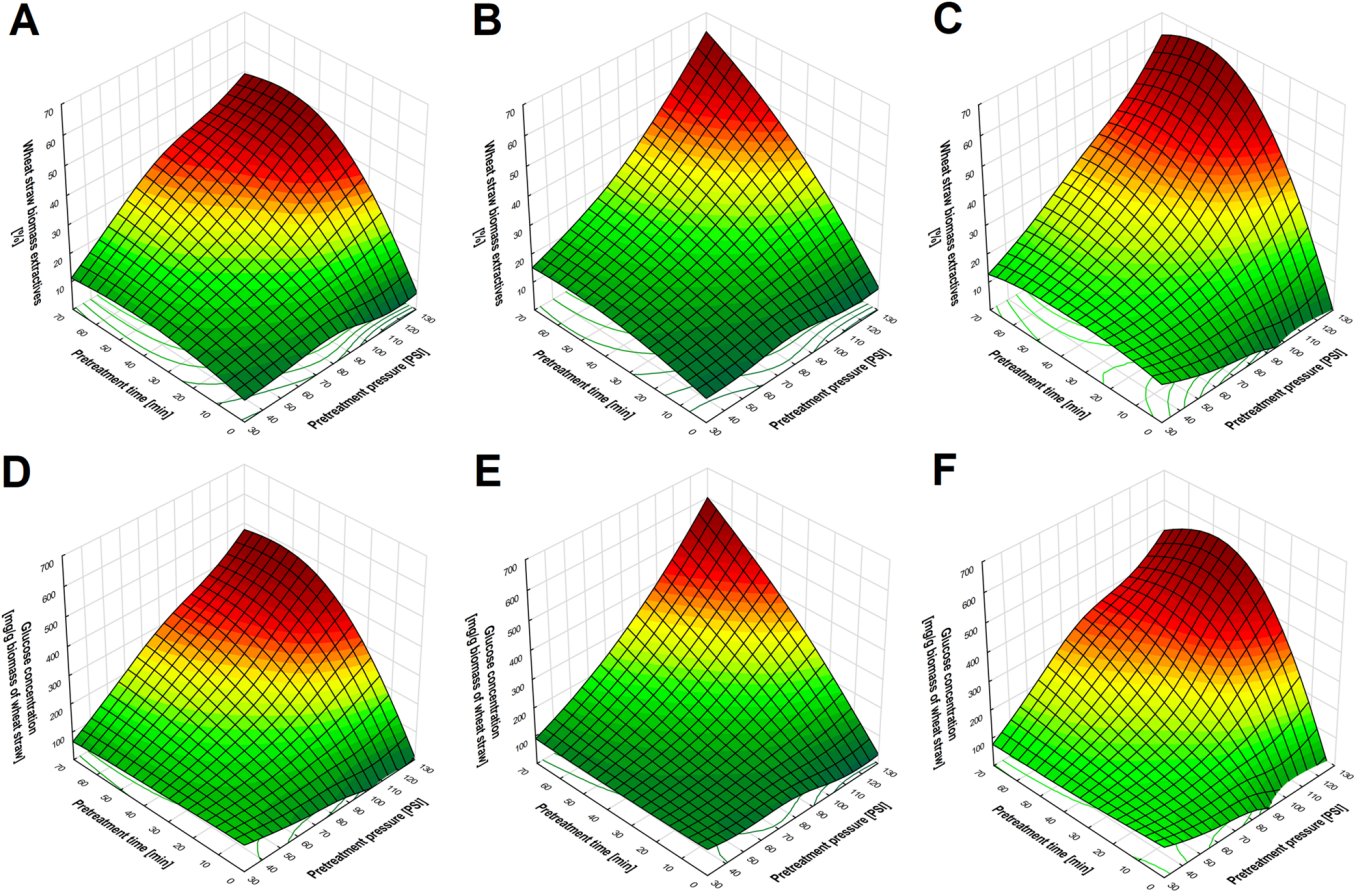
The effect of NaCS concentration ((**A,D**) 10% w/v NaCS; (**B,E**) 20% w/v NaCS; (**C,F**) 40% w/v NaCS), pretreatment time and pressure on biomass extractives and on glucose concentration obtained by 72 h of enzymatic hydrolysis of cellulose after microwave-assisted hydrotropic pretreatment of wheat straw.

**Figure 2:**
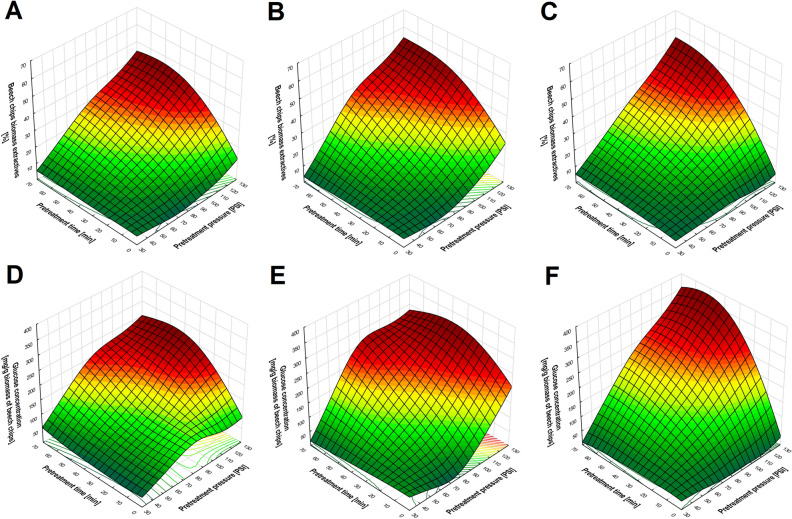
The effect of NaCS concentration ((**A,D**) 10% w/v NaCS; (**B,E**) 20% w/v NaCS; (**C,F**) 40% w/v NaCS), pretreatment time and pressure on biomass extractives and on glucose concentration obtained by 72 h of enzymatic hydrolysis of cellulose after microwave-assisted hydrotropic pretreatment of beech chips.

**Figure 3:**
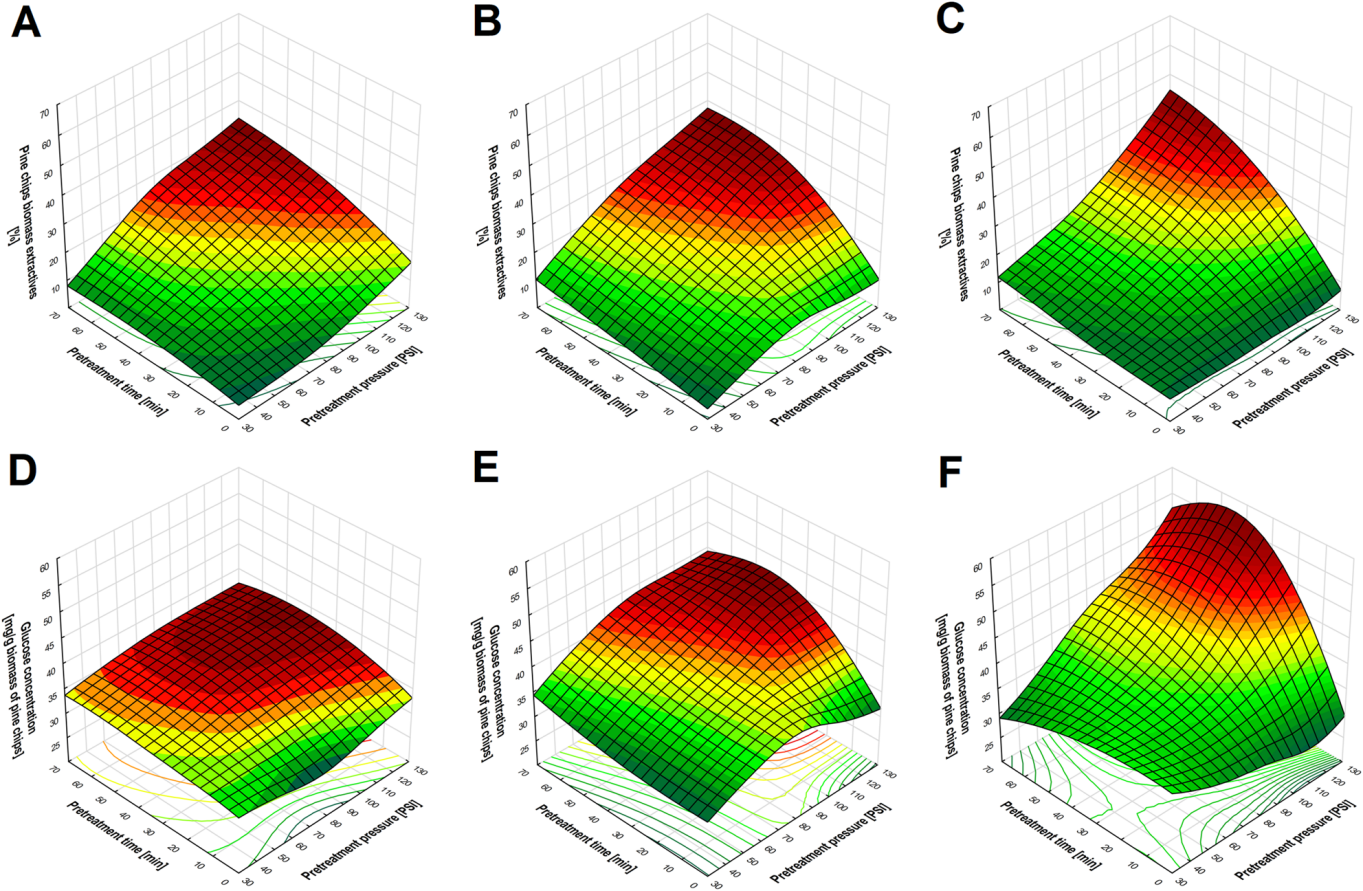
The effect of NaCS concentration ((**A,D**) 10% w/v NaCS; (**B,E**) 20% w/v NaCS; (**C,F**) 40% w/v NaCS), pretreatment time and pressure on biomass extractives and on glucose concentration obtained by 72 h of enzymatic hydrolysis of cellulose after microwave-assisted hydrotropic pretreatment of pine chips.

The effective use of the potential of lignocellulosic biomass depends on efficient biomass pretreatment, which should result in a high level of delignification. Lignins can be removed from biomass with the help of hydrotropes, which decrease surface tension and increase the effectiveness of the dissolution of phenolic compounds in aqueous solutions. An additional advantage of using hydrotrope in the form of sodium cumene sulfonate (NaCS) is the possibility of its recovery after the precipitation of lignins and its reuse after concentration. Evaluation of the effectiveness of the use of the microwave-assisted hydrotropic extraction of biomass requires detailed analysis of the impact of the changeable process conditions on the quantity of biomass extractives and lignocellulose composition after pretreatment. In view of differences in the composition of biomass of different origins, the tests covered softwood (pine chips), hardwood (beech chips) and non-wood (wheat straw) biomasses. A comparison of the degree of biomass loss generated as a result of microwave-assisted hydrotropic pretreatment showed that biomass extractives depend on the type of the biomass. The highest level of biomass loss as a result of pretreatment was determined for wheat straw, and the lowest for pine chips. The maximum level of extraction of biomass components (e.g. lignin, extractives) achieved as a result of microwave-assisted hydrotropic pretreatment was ca. 55% for wheat straw, ca. 45% for beech chips, and 35% for pine chips (Figs. 1, 2, 3). At constant NaCS concentration, regardless of the type of lignocellulosic biomass, increased loss of biomass was recorded as a result of pretreatment at increasing pressure and exposure. The highest loss of mass after microwave-assisted hydrotropic pretreatment was obtained at 117 PSI for 60 min, regardless of the biomass type. The effectiveness of the extraction of biomass components was also dependent on the NaCS concentration. At constant process conditions guaranteeing the highest level of mass loss (117 PSI, 60 min), the use of increasing hydrotrope concentration resulted in a ca. 5% increase in wheat straw biomass loss at NaCS concentrations of, subsequently, 10, 20, and 40% w/v (Fig. 1A–C). In the case of beech chips, the increase amounted to ca. 8% at analogical hydrotrope concentrations (Fig. 2A–C). The smallest increase in biomass loss was recorded for pine chips. It amounted to ca. 4% at 20% w/v NaCS (in relation to the use of 10% w/v NaCS) and ca. 2% at 40% w/v NaCS (in relation to the use of 20% w/v NaCS) (Fig. 3A–C).

### Analysis of lignocellulose composition and delignification efficiency

The content of structural polysaccharides is the main determinant deciding about the suitability of biomass as a source of carbon in biosynthesis processes. Because high lignin content is a factor limiting the effectiveness of cellulose and hemicellulose hydrolysis, it is significant that the pretreatment process enables effective delignification. In all biomass samples obtained after microwave-assisted hydrotropic pretreatment, the concentration of cellulose, hemicellulose and lignins was determined (Tables 1, 2, 3). In consistence with analytical methodology, constant weight of biomass after extraction, marked with different loss of mass as a result of pretreatment, was used for determination of components of lignocellulose. This resulted in the observed increase in the content of cellulose in the biomass. The higher the degree of extraction of biomass components (loss of mass as a result of extraction), the higher the content of cellulose (Table 1). This indicates an absence of cellulose degradation as a result of microwave-assisted pretreatment involving NaCS. The highest content of cellulose was identified in biomass samples subjected to microwave-assisted pretreatment involving 40% w/v NaCS at 117 PSI for 60 min (Table 1). Test results indicate that it is possible to achieve a high degree of purification of biomass from lignin fraction, which generates a very high content of cellulose in wheat straw samples at ca. 89% DW and beech chips at ca. 84% DW (Table 1). Microwave-assisted hydrotropic pretreatment also caused extraction of hemicellulose from biomass, lowering the content of this polysaccharide in the analysed samples (Table 2). The effectiveness of the extraction of hemicellulose from biomass is correlated with biomass extractives. The pretreatment method used enables an almost complete extraction of hemicellulose from wheat straw from the level of 29–30% DW and from pine chips (original content 12–13% DW) (Table 2). The extraction of lignins from biomass was less intense in comparison to hemicellulose. In the case of the use of 10% w/v NaCS, increased content of lignins in the biomass was recorded, while when 20% w/v NaCS was used, the content of lignins was constant regardless of the conditions of microwave-assisted pretreatment and biomass type (Table 3). The observed increase in lignin content in biomass results from the use of constant weights for determination of lignocellulose components. The lowering of lignin content in biomass was determined at as much as 40% w/v NaCS, although the level of extraction depended on the type of biomass. The lowest level of lignin extraction was recorded for softwood biomass (a ca. 5% decrease in content), while the greatest lowering of lignin content was observed for non-wood biomass (a ca. 45% decrease in content) (Table 3). The values present a relative content of lignins in the raw material, as they do not take into account the loss of biomass resulting from pretreatment. Hence, to determine the absolute effectiveness of delignification involving microwave-assisted hydrotropic pretreatment, the absolute content of lignins in the raw material, taking into account biomass extractives, was calculated. The resulting value was then subtracted from lignin content in the raw material before pretreatment to acquire a parameter enabling the determination of the absolute difference in lignin content in the raw material before and after pretreatment (Table 4). The calculated relative difference in lignin content in lignocellulosic raw material confirms the effectiveness of microwave-assisted hydrotropic delignification; however, the recorded effect is greater in view of a high level of extraction of biomass components. The greatest difference in absolute lignin content, regardless of the biomass type used, is recorded at 40% w/v NaCS, and 117 PSI for 60 min (Table 4). In these cases, the effectiveness of delignification reaches as much as 74% for wheat straw, ca. 58% for beech chips, and ca. 37% for pine chips. To determine the impact of the use of hydrotrope on the level of lignin extraction, the content of this component of lignocellulose was measured in various types of biomass after microwave-assisted pretreatment, in conditions guaranteeing the acquisition of the highest level of biomass extractives (117 PSI, 60 min) involving water (0% w/v NaCS) and 40% w/v NaCS (Table 5). The use of water only for microwave-assisted pretreatment caused an increase in the absolute lignin content in the raw material as a result of the extraction of water-soluble components from the biomass. The results show that the use of NaCS is the key factor enabling high effectiveness of delignification. As a result of using 40% w/v NaCS, absolute lignin content decreased by ca. 80% in wheat straw, by ca. 63% in beech chips, and by ca. 56% in pine chips (Table 5).

**Table 1:** Cellulose concentration in various types of biomass after microwave-assisted hydrotropic pretreatment.

Parameters of microwave-assisted hydrotropic pretreatment			Cellulose concentration [% DW] after pretreatment in various types of biomass		
NaCS concentration [% w/v]	Pressure [PSI]	Time [min]	Pine chips	Beech chips	Wheat straw
10	39	10	54.09 ± 0.26	62.12 ± 0.10	51.56 ± 0.15
	39	30	54.42 ± 0.13	61.99 ± 0.65	52.05 ± 0.23
	39	60	54.34 ± 0.46	60.75 ± 0.13	52.90 ± 0.05
	78	10	53.80 ± 0.44	64.18 ± 0.00	53.18 ± 0.49
	78	30	57.10 ± 0.69	63.05 ± 0.10	56.61 ± 0.26
	78	60	62.59 ± 0.46	68.82 ± 0.03	66.26 ± 0.02
	117	10	54.73 ± 0.13	64.72 ± 0.23	53.16 ± 0.00
	117	30	59.01 ± 0.64	73.61 ± 0.15	67.32 ± 0.36
	117	60	61.66 ± 0.05	76.07 ± 0.14	72.89 ± 0.36
20	39	10	48.42 ± 0.10	61.50 ± 0.00	51.64 ± 0.03
	39	30	50.58 ± 0.21	61.81 ± 0.10	53.01 ± 0.05
	39	60	50.76 ± 0.28	62.12 ± 0.21	54.78 ± 0.08
	78	10	53.98 ± 0.31	61.38 ± 0.23	51.61 ± 0.10
	78	30	55.22 ± 0.46	64.37 ± 0.12	54.53 ± 0.03
	78	60	59.34 ± 0.21	73.03 ± 0.09	61.04 ± 0.05
	117	10	53.47 ± 0.10	69.87 ± 0.05	52.39 ± 0.05
	117	30	60.79 ± 0.05	80.13 ± 0.10	64.42 ± 0.03
	117	60	63.49 ± 0.07	84.12 ± 0.05	81.18 ± 0.21
40	39	10	52.49 ± 0.15	61.72 ± 0.26	51.18 ± 0.28
	39	30	52.16 ± 0.03	61.38 ± 0.08	55.43 ± 0.00
	39	60	53.73 ± 0.21	61.04 ± 0.05	53.52 ± 0.15
	78	10	53.42 ± 0.15	61.95 ± 0.03	50.38 ± 0.10
	78	30	55.58 ± 0.15	64.11 ± 0.24	57.44 ± 0.05
	78	60	59.47 ± 0.24	69.94 ± 0.02	66.17 ± 0.17
	117	10	56.90 ± 0.18	63.39 ± 0.07	53.80 ± 0.03
	117	30	64.34 ± 0.05	74.12 ± 0.15	77.20 ± 0.04
	117	60	68.87 ± 0.15	83.96 ± 0.10	89.17 ± 0.05

**Table 2:** Hemicellulose concentration in various types of biomass after microwave-assisted hydrotropic pretreatment.

Parameters of microwave-assisted hydrotropic pretreatment			Hemicellulose concentration [% DW] after pretreatment in various types of biomass		
NaCS concentration [% w/v]	Pressure [PSI]	Time [min]	Pine chips	Beech chips	Wheat straw
10	39	10	12.43 ± 0.05	18.85 ± 0.01	29.07 ± 0.10
	39	30	13.09 ± 0.43	20.12 ± 0.45	28.96 ± 0.02
	39	60	12.78 ± 0.30	20.02 ± 0.05	27.49 ± 0.10
	78	10	11.69 ± 0.35	16.17 ± 0.11	28.43 ± 0.19
	78	30	7.78 ± 0.06	17.82 ± 0.09	24.11 ± 0.30
	78	60	2.79 ± 0.31	9.74 ± 0.02	11.47 ± 0.05
	117	10	5.97 ± 0.19	15.49 ± 0.06	28.45 ± 0.21
	117	30	1.76 ± 0.12	5.67 ± 0.09	10.88 ± 0.15
	117	60	0.56 ± 0.15	0.83 ± 0.10	2.44 ± 0.05
20	39	10	13.44 ± 0.06	21.53 ± 0.01	29.90 ± 0.04
	39	30	12.61 ± 0.16	22.72 ± 0.05	29.04 ± 0.21
	39	60	11.39 ± 0.17	22.15 ± 0.11	28.47 ± 0.02
	78	10	10.66 ± 0.15	22.11 ± 0.04	31.23 ± 0.00
	78	30	8.22 ± 0.26	18.57 ± 0.22	27.28 ± 0.24
	78	60	4.69 ± 0.25	10.25 ± 0.16	20.70 ± 0.10
	117	10	10.25 ± 0.04	12.79 ± 0.00	28.27 ± 0.26
	117	30	3.51 ± 0.10	3.46 ± 0.36	16.98 ± 0.04
	117	60	0.16 ± 0.06	0.10 ± 0.00	2.07 ± 0.21
40	39	10	12.35 ± 0.31	21.47 ± 0.10	29.52 ± 0.24
	39	30	13.25 ± 0.17	22.16 ± 0.32	25.70 ± 0.14
	39	60	11.19 ± 0.05	21.88 ± 0.05	28.80 ± 0.14
	78	10	12.32 ± 0.26	22.16 ± 0.20	27.04 ± 0.27
	78	30	9.84 ± 0.16	19.23 ± 0.24	26.36 ± 0.00
	78	60	6.96 ± 0.05	13.51 ± 0.02	15.97 ± 0.09
	117	10	11.30 ± 0.06	20.82 ± 0.13	27.68 ± 0.22
	117	30	4.06 ± 0.15	9.83 ± 0.36	8.25 ± 0.21
	117	60	0.21 ± 0.00	2.41 ± 0.15	0.11 ± 0.00

**Table 3:** Lignin concentration in various types of biomass after microwave-assisted hydrotropic pretreatment.

Parameters of microwave-assisted hydrotropic pretreatment			Lignin content [% DW] after pretreatment in various types of biomass		
NaCS concentration [% w/v]	Pressure [PSI]	Time [min]	Pine chips	Beech chips	Wheat straw
10	39	10	28.88 ± 0.31	11.95 ± 0.21	9.32 ± 0.15
	39	30	28.81 ± 0.35	12.05 ± 0.10	9.33 ± 0.05
	39	60	29.00 ± 0.15	12.56 ± 0.01	9.53 ± 0.05
	78	10	29.07 ± 0.19	12.88 ± 0.10	9.27 ± 0.20
	78	30	28.86 ± 0.53	12.77 ± 0.00	9.53 ± 0.05
	78	60	29.62 ± 0.26	14.58 ± 0.04	10.66 ± 0.15
	117	10	33.05 ± 0.22	13.75 ± 0.06	9.27 ± 0.10
	117	30	33.29 ± 0.33	14.89 ± 0.16	11.23 ± 0.10
	117	60	34.31 ± 0.21	16.03 ± 0.15	12.88 ± 0.21
20	39	10	33.12 ± 0.15	12.16 ± 0.10	8.40 ± 0.05
	39	30	31.99 ± 0.26	11.69 ± 0.05	8.09 ± 0.15
	39	60	32.62 ± 0.34	11.54 ± 0.21	8.25 ± 0.00
	78	10	30.96 ± 0.05	11.70 ± 0.16	8.45 ± 0.00
	78	30	31.56 ± 0.31	12.27 ± 0.01	8.45 ± 0.11
	78	60	32.19 ± 0.05	13.14 ± 0.15	8.81 ± 0.15
	117	10	32.09 ± 0.05	12.01 ± 0.16	8.35 ± 0.10
	117	30	31.92 ± 0.05	11.81 ± 0.16	8.45 ± 0.10
	117	60	33.19 ± 0.02	12.00 ± 0.15	8.24 ± 0.10
40	39	10	28.90 ± 0.05	12.12 ± 0.16	9.12 ± 0.15
	39	30	28.76 ± 0.09	11.96 ± 0.20	8.40 ± 0.15
	39	60	28.41 ± 0.15	12.47 ± 0.00	7.93 ± 0.21
	78	10	28.64 ± 0.00	11.70 ± 0.06	8.50 ± 0.05
	78	30	28.64 ± 0.21	11.85 ± 0.10	7.88 ± 0.05
	78	60	28.14 ± 0.19	11.03 ± 0.01	7.67 ± 0.16
	117	10	28.24 ± 0.22	11.29 ± 0.16	7.73 ± 0.11
	117	30	28.02 ± 0.00	11.43 ± 0.10	6.64 ± 0.06
	117	60	27.76 ± 0.05	9.63 ± 0.15	5.05 ± 0.00

**Table 4:** Change in absolute lignin concentration after microwave-assisted hydrotropic pretreatment.

Parameters of microwave-assisted hydrotropic pretreatment			Change in absolute lignin concentration [%] after pretreatment compared to biomass without pretreatment		
NaCS concentration [% w/v]	Pressure [PSI]	Time [min]	Pine chips	Beech chips	Wheat straw
10	39	10	− 2.61	− 7.95	− 3.63
	39	30	− 5.46	− 8.30	− 7.64
	39	60	− 6.60	− 6.86	− 7.52
	78	10	− 5.47	− 3.57	− 9.08
	78	30	− 11.84	− 13.32	− 14.12
	78	60	− 17.96	− 12.29	− 17.89
	117	10	− 1.65	− 8.61	− 12.25
	117	30	− 9.14	− 19.52	− 20.14
	117	60	− 13.44	− 21.35	− 19.27
20	39	10	+ 11.47	− 6.07	− 13.58
	39	30	+ 4.99	− 11.21	− 20.59
	39	60	+ 2.97	− 13.94	− 21.45
	78	10	− 5.23	− 13.22	− 16.91
	78	30	− 7.42	− 17.44	− 22.98
	78	60	− 13.16	− 27.59	− 28.12
	117	10	− 4.32	− 26.96	− 19.60
	117	30	− 18.25	− 41.48	− 34.82
	117	60	− 21.54	− 48.36	− 52.66
40	39	10	− 4.17	− 6.70	− 8.80
	39	30	− 5.81	− 9.73	− 18.77
	39	60	− 10.37	− 7.73	− 24.23
	78	10	− 7.28	− 14.55	− 13.79
	78	30	− 13.75	− 20.38	− 30.21
	78	60	− 20.28	− 34.32	− 41.13
	117	10	− 12.21	− 22.49	− 25.43
	117	30	− 26.35	− 39.78	− 58.72
	117	60	− 36.58	− 57.68	− 74.08

**Table 5:** Lignin concentration in various types of biomass after microwave-assisted pretreatment with water (0% w/v NaCS) and 40% w/v NaCS.

Type of biomass	Pine chips	Beech chips	Wheat straw
Lignin concentration [% DW] after pretreatment with 0% w/v NaCS (water), 117 PSI, 60 min	51.19 ± 0.24	17.24 ± 0.15	17.55 ± 0.27
Absolute lignin concentration [% DW] after pretreatment with 0% w/v NaCS (water), 117 PSI, 60 min	40.12 ± 0.19	14.10 ± 0.12	10.99 ± 0.17
Lignin concentration [% DW] after pretreatment with 40% w/v NaCS, 117 PSI, 60 min	27.76 ± 0.05	9.63 ± 0.15	5.05 ± 0.00
Absolute lignin concentration [% DW] after pretreatment with 40% w/v NaCS, 117 PSI, 60 min	17.52 ± 0.03	5.18 ± 0.08	2.24 ± 0.00
Change in absolute lignin concentration [%] after pretreatment with 40% w/v NaCS compared to pretreatment with 0% w/v NaCS (water)	− 56.33	− 63.26	− 79.62

### Cellulose hydrolysis

The concentration of glucose obtained as a result of enzymatic hydrolysis of cellulose is strongly correlated with biomass extractives. A higher concentration of glucose as a result of enzymatic hydrolysis was achieved from the raw material marked by a high level of loss of biomass components and a high content of cellulose in the biomass, which is related to the already discussed effect of the absence of cellulose extraction during microwave-assisted hydrotropic pretreatment. Obtaining the highest concentration of glucose as a result of hydrolysis of a given type of biomass subjected to microwave-assisted hydrotropic pretreatment has always been conditioned by the use of 40% w/v NaCS at 117 PSI for 60 min. As a result of enzymatic hydrolysis of non-wood (wheat straw) and hardwood (beech chips) biomass, a high concentration of glucose was achieved at, respectively, 463.27 ± 11.25 mg/g of biomass (hydrolysis yield 46.76 ± 1.14%) and 327.70 ± 22.15 mg/g of biomass (hydrolysis yield 35.13 ± 2.37%) (Figs. 1D–F, 2D–F). The highest resistance to enzymatic hydrolysis involving cellulases was determined for pine chip (softwood) biomass, which yielded a maximum glucose concentration at 50.77 ± 0.75 mg/g of biomass (hydrolysis yield 6.63 ± 0.10%) (Fig. 3D–F). The processed wheat straw and beech chip biomass obtained as a result of microwave-assisted hydrotropic pretreatment was marked by the highest cellulose content and a high susceptibility to enzymatic hydrolysis (effect of a high level of delignification), which speaks for the use of these raw materials as a source of carbon in microbiological biosynthesis processes. From among the analysed sources of biomass, the lowest susceptibility to enzymatic hydrolysis of cellulose was recorded for pine chips, which is related to the lower level of delignification obtained.

## Discussion

Delignification of plant biomass is one of the key elements of the pretreatment of lignocellulose, determining its effective use as a source of carbon in biosynthesis processes. The removal of lignins from biomass facilitates the delivery of the process of the enzymatic hydrolysis of structural polysaccharides, simultaneously contributing to a more effective use of biomass, e.g. in the production of cellulose ethanol. However, the lignin fraction is hard to extract due to its structure and a strongly hydrophobic nature of phenolic compounds^1,9^. Compounds which effectively remove lignins from biomass include organic solvents (ethanol), deep eutectic solvents and hydrotropes^18,34^. To intensify the process of delignification, physical factors are often additionally employed, including conventional heating or microwave radiation allowing an increase in temperature and pressure^30^. The present study focused on one such solution, assessing the impact of the combined use of hydrotrope in the form of sodium cumene sulfonate and microwave radiation on increasing the effectiveness of the delignification of various types of biomass (softwood, hardwood and non-wood). The results clearly indicate the effectiveness of the suggested solution; however, the ultimate effectiveness of the proposed method closely depends on the process conditions and type of biomass. Pressure is one of the key parameters of microwave hydrotropic delignification and its increase, as well as, in consequence, the increase in temperature, intensifies the removal of lignins. Higher pressure and temperature are tantamount to the lower density and viscosity of the solution of hydrotropes and their higher ability to penetrate structures of lignocellulosic biomass. Other authors also confirm the impact of temperature on the effectiveness of delignification and the loss of biomass components. An increase of temperature of NaCS extraction from 40 to 80 °C with the help of conventional heating causes an increased loss of rice biomass by ca. 10%^33^. In turn, the use of 117 PSI and 20% w/v NaCS enables a high level of component extraction (in excess of 65%) from maize distillery stillage—higher than in the present study, which results from the nature of the lignocellulosic biomass. Waste distillery stillage biomass is a by-product of the technology of production of first generation ethanol involving starch raw materials. Some technologies are marked by barothermal processing of the raw material (5–6 atm, 50 min) affecting the structure of lignocelulose^35^. The effectiveness of the delignification of sugarcane bagasse using sodium xylene sulfonate (NaXS) also increases by ca. 40% along with the temperature increase from 60 to 120 °C^20^. It was also observed that when using NaXS in the delignification of wheat straw involving conventional heating, the loss of lignin content in the biomass increased by ca. 5% DW at a temperature increasing from 120 to 180 °C^22^. Similar tendencies were recorded when using deep eutectic solvents. The use of a mixture of choline chloride and monoethanolamine in lignin extraction from wheat straw generated an increased effectiveness of delignification to ca. 98% at 130 °C, although the process lasted for as many as 13 h^34^. The effectiveness of the delignification of birchwood involving choline chloride and formic acid also increased along with the use of a higher temperature and reached the highest value at ca. 80% in 130 °C^25^. Presented study determined a positive effect of prolonged microwave-assisted hydrotropic pretreatment on the degree of the delignification of various types of biomass. In other authors’ studies, in which a conventional way of heating of biomass was used, this effect is minimal. It was discovered that by prolonging the duration of the process of the delignification of birch or pine wood using NaXS from 30 to 120 min, the lignin content decreased by as little as ca. 2–2.5% DW^36,37^. In the case of the delignification of wheat straw using NaXS, extending the time of lignin extraction from 30 to 120 min decreased the lignin content by only ca. 3% DW^22^. The present study shows that by prolonging the duration of microwave delignification from 10 to 60 min, the effectiveness of the process can increase by ca. 50% if wheat straw is used as the raw material (117 PSI, 40% w/v NaCS). Other authors showed that extending the duration of lignin removal from sugarcane bagasse from 60 to 240 min using conventional heating and 40% w/w NaXS increases the effectiveness of the delignification process by ca. 40%. This clearly indicates a higher effectiveness reached over a shorter time when using the process involving microwave radiation^20^. The impact of the duration of the delignification process on the generated results also depends on the type of biomass used. A similar relation as in presented study was observed when using the biomass of eucalyptus, cotton stalks, wood waste (containing 80% of birchwood and 20% of beechwood), and reed as a source of lignocellulose. Hydrotropic pretreatment using NaXS at 160 °C for 90 min allowed ca. 6.5% DW reduction of lignins in eucalyptus biomass^18^. A similar level of lignin content reduction (ca. 7% DW) was achieved after pretreatment of reed biomass at 30% w/v NaXS, 160 °C, for 60 min. 34 A higher level of lignin content reduction (ca. 13% DW) was achieved using 40% w/v NaXS in the pretreatment of wood waste^38^. Also the reduction of lignin content in cotton stalks was highly effective (reduction by ab. 11% DW) using 30% w/v NaCS at 121 °C for 120 min^21^. Also the use of a phenoxyethanol/acid mixture in changeable proportions enables an effective delignification of bamboo biomass. The effectiveness of the delignification of bamboo biomass may reach as much as ca. 90% when a 4:1 phenoxyethanol/acid mixture is used at 120 °C^39^. Our study demonstrates the possibility of obtaining the highest concentration of glucose as a result of the enzymatic hydrolysis of biomass marked by the highest level of delignification. The achieved high concentration of glucose from one gram of biomass results from the high content of cellulose and its susceptibility to enzymatic hydrolysis using cellulases. A high correlation between the level of delignification and the effectiveness of hydrolysis (at R2 = 0.94) was also determined in the case of the use of bamboo biomass after pretreatment involving phenoxyethanol-acid in changeable proportions^39^. In turn, the reduction of the content of lignins in eucalyptus biomass using sodium xylenesulphonate contributes to an intensification of the enzymatic hydrolysis of cellulose. The effectiveness of glucose from eucalyptus biomass after enzymatic hydrolysis increases from ca. 30 to ca. 70% when an effective manner of delignification, enabling lignin content reduction by ca. 6%, is used^18^. Apart from our own earlier studies, relevant literature does not present any research describing the effects of the simultaneous use of hydrotropes and microwave radiation in the delignification of lignocellulosic biomass. Our research clearly shows the suitability of microwave-assisted hydrotropic pretreatment in the process of lignin removal from various types of biomass; however, the effectiveness of a given pretreatment method depends on the type of biomass, its structure and process parameters. In comparison to other authors’ studies, the use of microwave-assisted pretreatment involving NaCS enables the achievement of a high level of lignin removal in a relatively short time. Additionally, lignocellulosic biomass after being pre-treated is susceptible to enzymatic hydrolysis, as manifested by the generation of a high concentration of glucose from one gramme of the thus-prepared biomass.

## Materials and methods

### Raw materials

Lignocellulosic biomass in the form of pine chips, beech chips and wheat straw was used in this study. The wood waste samples originated from a sawmill (Wudzyń, Kujawsko-Pomorskie, Poland), while the wheat straw from a farm (Radzicz, Kujawsko-Pomorskie, Poland). All biomass samples had a dry weight (DW) content of 95.05 ± 0.52%. Lignocellulosic biomass samples were initially ground using a hammer mill and sieved through a sieve with an aperture size of 1 mm. The lignocellulose components included: cellulose 51.23 ± 0.13% DW, hemicellulose 10.47 ± 0.05% DW, lignins 27.63 ± 0.12% DW for pine chips; cellulose 59.95 ± 0.26% DW, hemicellulose 17.75 ± 0.36% DW, lignins 12.25 ± 0.11% DW for beech chips; and cellulose 45.54 ± 0.05% DW, hemicellulose 21.64 ± 0.03% DW, lignins 8.65 ± 0.11% DW for wheat straw.

### Hydrotrope

Sodium cumene sulfonate (NaCS) in the form of 40% w/v of Stepanate SCS 40 by Stepan Company (Northfield, Illinois, United States) was used as a hydrotrope in the process of delignification.

### Cellulolytic enzyme

Presented study into the susceptibility of various types of lignocellulosic biomass after pretreatment on the enzymatic hydrolysis of cellulose involved Cellic CTec2 (Novozymes, Franklinton, NC, USA) containing a blend of cellulolytic enzymes with the activity of 75 FPU/mL. The activity of cellulase expressed in FPU (filter paper units) was determined in consistence with the Technical Report NREL/TP-510-42628. The preparation was used at a dosage of 5 FPU/g DW of biomass, as recommended by the manufacturer, at pH 5.5 and 50 °C.

### Microwave-assisted hydrotropic pretreatment

The study analysed the impact of changeable parameters of microwave-assisted pretreatment involving various concentrations of NaCS on changes of the composition of lignocellulose in the types of biomass specified above as well as their susceptibility to subsequent enzymatic hydrolysis. Microwave-assisted hydrotropic pretreatment was conducted at 600 W constant power of a microwave generator, since our previous studies did not identify the impact of this parameter on the effectiveness of pretreatment^32^. The concentration of biomass in the solution during extraction was kept at the constant level of 5.3%. Changeable pretreatment parameters included different concentrations of NaCS (10, 20, 40% v/w), pressure (39, 78, 117 PSI) and exposure time (10, 30, 60 min), which made it possible to carry out pretreatment for each of the three raw materials in 27 research variants. The execution of each research variant was commenced with the weighing of ca. 2.5 g of biomass and drying it at 130 °C to a constant mass (the thus-obtained weights were recorded). Extraction was carried out using five repetitions. Each weight was then transferred to HP-500 plus Teflon vessels and 45 mL of NaCS solution was added. Microwave delignification was conducted in the microwave digestion system Mars 5 (CEM Corporation) enabling pretreatment under controlled pressure, time and microwave generator power conditions. After pretreatment, the solution was trickled under lowered pressure and rinsed with 250 mL of 60 °C water. The resulting biomass was subsequently dried to a constant mass at 130 °C. Biomass extractives were calculated from the difference between the weight of biomass before and after pretreatment. To determine the impact of the use of the hydrotrope on the level of lignin extraction, microwave-assisted pretreatment using water (0% w/v NaCS) in the conditions guaranteeing the highest obtained level of extraction of biomass components, i.e. 60 min, 117 PSI, was also carried out. The thus-acquired biomass was kept until an analysis of lignocellulose composition and susceptibility to enzymatic hydrolysis was conducted.

### Enzymatic hydrolysis

All the kinds of biomass which underwent pretreatment as described above were subjected to enzymatic hydrolysis to determine the quantity of glucose acquired from 1 g of biomass over 72 h of hydrolysis using cellulolytic enzymes. The process of hydrolysis was started with the placing of 1 g DW of pretreated biomass into a 100 mL Erlenmeyer flask and adding 25 mL of 0.05 M of acetate buffer with pH 5.5 (biomass concentration in the solution was 4%). The acquired suspension was placed in a 50 °C water bath and 67 µL of Cellic CTec2 (5 FPU/g DW of biomass) was added. The process of enzymatic hydrolysis was carried out at 50 °C, with shaking at 70 rpm, for 72 h. An analysis of the glucose concentration in the solution was conducted every 24 h using the method involving HPLC. Based on the concentration of glucose, the yield of the hydrolysis process was calculated from the formula:

$${\text{Yield of hydrolysis}} [\% ] = \frac{{{\text{C}}_{{{\text{Glu}}}} }}{{1.111 \times {\text{C}}_{{{\text{Cel}}}} \times {\text{C}}_{{{\text{Biom}}}} }} \times 100,$$

where C_Glu_ is the concentration of glucose (grams per liter) in the sample, C_Cel_ is the cellulose content in biomass (per 1 g of dry weight), C_Biom_ is the initial biomass content (grams per liter)^32^.

### Analytical methods

Biomass before and after pretreatment was analysed for the content of cellulose, hemicellulose and lignins to determine the impact of parameters of microwave-assisted hydrotropic pretreatment on lignocellulose composition. The determination of lignocellulose components was carried out using Fibertec 8000 by FOSS (Hilleroed, Denmark) in consistence with international standards ISO13906:2008 and ISO16472:2006. The analysis involved the determination of Neutral Detergent Fiber (NDF), Acid Detergent Fiber (ADF) and Acid Detergent Lignin (ADL) in the biomass with the subsequent calculation of the content of cellulose from the difference between ADF and ADL, and hemicellulose from the difference between NDF and ADF; the lignin content was equal to the ADL^40^. The absolute lignin content in pretreated biomass was calculated on the basis of biomass extractives and the content of lignins, using the following formula:

$$\text{Absolute lignin concentration } = \frac{\text{Lignin concentration}}{\left(\frac{1}{\text{Biomass concentration}}\right)},$$

where the biomass concentration is understood as the actual amount of biomass used for the determination of the lignin content taking into account the level of biomass extractives resulting from microwave extraction. The Biomass Concentration was calculated using the following formula:

$$\text{Biomass }{\text{c}}{\text{oncentration}} \, \text{=}\frac{1}{\left({1}-\left(\frac{\text{Biomass extractives }}{100}\right)\right)}.$$

The drying of lignocellulosic biomass samples and determination of dry mass in samples (DW) took place using weighting dryer by RadWag, model MA 50.R1NS. Glucose concentration (g/L) was determined in the experimental medium after 24, 48 and 72 h of enzymatic hydrolysis using high-performance liquid chromatographer equipped with refractometric detector (HPLC-RID) model 1260 by Agilent Technologies (Palo Alto, CA, USA). The determination was commenced with centrifugation of the suspension at 5000 ×*g* for 5 min at 15 °C (260R, MPW, Poland) and its ten-fold dilution in the mobile phase for HPLC, i.e. in 5 mM H_2_SO_4_. The solution was then filtered through a membrane filter with PES with the aperture size of 0.45 µm. Chromatographic division was performed using Hi-Plex H column (Agilent Technologies, Palo Alto, CA, USA) equipped with a guard column at 60 °C with the isocratic flow of the mobile phase at 0.6 mL/min. Detection was carried out at 50 °C. Quantitative determination was performed using the external standards method (ESTD) in the form of calibration curves of glucose solutions^32^. All experiments and methods were performed in accordance with relevant guidelines and regulations.

### Statistical analysis

All laboratory analyses were performed in triplicate. Statistical analysis (analysis of variance, determination of standard deviation—SD, response surface methodology—RSM) was carried out using the TIBCO Software Inc. Statistica ver. 13 (Palo Alto, CA, USA). An ANOVA test and an HSD Tukey’s test were applied at the significance level of α < 0.05.

### Compliance with ethics requirements

This article does not contain any studies on human or animal subjects.

## Conclusions

Microwave-assisted hydrotropic pretreatment using NaCS is an effective method of the delignification of hardwood and non-wood biomasses. Conclusions facilitating the highest delignification of raw materials used include NaCS concentration at 40% w/v, pressure of 117 PSI and duration of 60 min. Under these conditions it is possible to obtain biomass with significantly lower lignin content and an increased content of cellulose susceptible to enzymatic hydrolysis. The obtained biomass after microwave-assisted hydrotropic pretreatment is a potential source of carbon that can be used in microbiological biosynthesis processes. Further research is planned to identify the impact of microwave-assisted hydrotropic pretreatment on changes in the structure of biomass of various origins using XRD, NMR and SEM techniques. The development of a highly efficient method of delignification will be the basis for research into highly efficient enzymatic hydrolysis in optimum process conditions and the use of hydrolysates in the process of the biosynthesis of cellulosic ethanol.

## Data Availability

The datasets generated and analysed during the current study are available in the Zenodo repository (https://doi.org/10.5281/zenodo.5833772).
